# Structural Brain Changes in Blepharospasm: A Cortical Thickness and Diffusion Tensor Imaging Study

**DOI:** 10.3389/fnins.2020.543802

**Published:** 2020-10-29

**Authors:** Yaomin Guo, Kangqiang Peng, Zilin Ou, Linchang Zhong, Ying Wang, Chuanmiao Xie, Jinsheng Zeng, Weixi Zhang, Gang Liu

**Affiliations:** ^1^Department of Neurology, The First Affiliated Hospital, Sun Yat-sen University, Guangdong Provincial Key Laboratory of Diagnosis and Treatment of Major Neurological Diseases, National Key Clinical Department and Key Discipline of Neurology, Guangzhou, China; ^2^Department of Medical Imaging, Sun Yat-sen University Cancer Center, State Key Laboratory of Oncology in Southern China, Collaborative Innovation Center for Cancer Medicine, Guangzhou, China

**Keywords:** blepharospasm, cortical thickness, diffusion tensor imaging, fractional anisotropy, local diffusion homogeneity

## Abstract

White matter abnormalities in blepharospasm (BSP) have been evaluated using conventional intra-voxel metrics, and changes in patterns of cortical thickness in BSP remain controversial. We aimed to determine whether local diffusion homogeneity, an inter-voxel diffusivity metric, could be valuable in detecting white matter abnormalities for BSP; whether these changes are related to disease features; and whether cortical thickness changes occur in BSP patients. Diffusion tensor and structural magnetic resonance imaging were collected for 29 patients with BSP and 30 healthy controls. Intergroup diffusion differences were compared using tract-based spatial statistics analysis and measures of cortical thickness were obtained. The relationship among cortical thickness, diffusion metric in significantly different regions, and behavioral measures were further assessed. There were no significant differences in cortical thickness and fractional anisotropy between the groups. Local diffusion homogeneity was higher in BSP patients than controls, primarily in the left superior longitudinal fasciculus, corpus callosum, left posterior corona radiata, and left posterior thalamic radiata (*P <* 0.05, family-wise error corrected). The local diffusion homogeneity values in these regions were positively correlated with the Jankovic rating scale (*r*_*s*_ = 0.416, *P* = 0.031) and BSP disability index (*r*_*s*_ = 0.453, *P* = 0.018) in BSP patients. These results suggest that intra- and inter-voxel diffusive parameters are differentially sensitive to detecting BSP-related white matter abnormalities and that local diffusion homogeneity might be useful in assessing disability in BSP patients.

## Introduction

Blepharospasm (BSP) is a common focal dystonia characterized by excessive involuntary contractions of the orbicularis oculi muscle without significant morphological brain abnormalities evaluated by conventional imaging techniques (Defazio et al., 2017); however, the etiology, pathophysiology, and symptom progression of BSP remain unclear. For other types of focal dystonia, studied using neuroimaging and neurophysiological techniques have highlighted the central role of abnormalities in circuits connecting the sensorimotor cortex to the basal ganglia, brainstem, and cerebellum (Baker et al., 2003; Schmidt et al., 2003; Martino et al., 2011; Berman et al., 2018). Notably, gray matter alterations in the basal ganglia, sensorimotor cortex, and cerebellum have been widely reported in BSP patients (Etgen et al., 2006; Obermann et al., 2007; Martino et al., 2011; Suzuki et al., 2011; Horovitz et al., 2012). We speculate that white matter (WM) changes may occur in BSP patients, especially in the fiber tracts that connect the basal ganglia, brainstem, cerebellum, and sensorimotor cortex; however, findings from diffusion tensor imaging (DTI) studies have been inconsistent. Two DTI studies, one using a region of interest (ROI)-based method (Fabbrini et al., 2008) and the other using a whole-brain method (Berman et al., 2018), were conducted in BSP patients, but no significant changes were observed in fractional anisotropy (FA) in the WM tracts. Regardless, significant FA reductions were detected only in the left anterior lobe of the cerebellum in another DTI study, which was negatively correlated with disease severity in BSP patients (Yang et al., 2014).

Diffusion indices, such as FA and mean diffusivity (MD), reflect diffusion properties solely within voxels. Recently, Gong (2013) proposed local diffusion homogeneity (LDH), an inter-voxel measurement to study inter-voxel features. This metric obtains the overall coherence of water diffusion within a neighborhood by utilizing the signals from the whole set of gradient directions. Liu et al. (2017) found a loss in LDH, but not FA, in the ipsilesional corticospinal tract in the acute phase of subcortical infarction using tract-based spatial statistics (TBSS), which could successfully predict the resolution of motor impairment within 3 months after stroke. A lower LDH was also found in the anterior corpus callosum (CC) in patients with mesial temporal lobe epilepsy compared to controls, but no significant changes of FA and MD were detected in this region. Moreover, the LDH values of the anterior CC could accurately discriminate patients from controls (Liu et al., 2016). Liang et al. (2019) also found that although there is overlap in the WM areas showing significant changes in FA and LDH between patients with type two diabetes mellitus and healthy controls, diabetic patients demonstrated higher LDH than controls in unique regions including the left temporal pole and pons. Speculatively, LDH may be less sensitive to the degree of myelination, but more sensitive to the fiber coherence than FA in specific WM regions showing significant changes of LDH but no FA between groups. Thus, LDH is complementary to traditional DTI parameters and can provide additional insights into WM variability between subjects; however, it is unclear whether LDH measurements can provide valuable information regarding WM abnormality detection for BSP, and if these changes are related to disease features.

Two cortical thickness (CT) studies reported widespread cortical atrophy in motor, sensory, and visual processing regions in BSP patients compared to healthy controls (Hanganu et al., 2016; Vilany et al., 2017); however, an uncorrected *P*-value was used to identify regions with significant CT changes. Therefore, whether CT changes occur in BSP patients remains unknown. Changes in gray matter volume have been found in widespread cortical areas using voxel-based morphometry (VBM) in BSP patients (Etgen et al., 2006; Obermann et al., 2007; Martino et al., 2011; Suzuki et al., 2011; Horovitz et al., 2012). Changes in CT, surface area, and gyrification may mediate changes in gray matter volume (Kong et al., 2015); however, it is unclear whether these changes can be attributed to CT changes.

In the present study, we aimed to address these gaps in the literature by investigating patterns of gray matter and WM microstructural alterations in BSP patients based on CT, FA, and LDH, and correlate these changes with behavioral measures. We hypothesized that compared to FA and LDH would detect additional WM alterations, which may be useful for the assessment of disability in BSP patients.

## Materials and Methods

### Participants

The present research was approval by the ethical committee of the First Affiliated Hospital of Sun Yat-sen University ([2020]323). BSP diagnosis was made according to the published standard criteria by a senior neurologist (WZ) from our outpatient clinic for movement disorders (Albanese et al., 2013). Patients who received botulinum toxin (BoNT) treatment were recruited at least 3 months post-injection. Exclusion criteria were the presence of traumatic brain injury, stroke, Alzheimer’s disease, epilepsy, Parkinson’s disease, evidence of possible anxiety (Hamilton Anxiety Scale score >14; Hamilton, 1959), and history of alcohol or drug abuse; presence of significant clinical comorbidities; history of exposure to medications known to induce dystonia, abnormalities at neuroimaging, and known causes of secondary dystonia; and/or medical implants that were contraindications for cerebral magnetic resonance imaging (MRI). We also recruited 30 healthy participants matched for age and handedness. All participants were right-handed. Written informed consent was provided by each subject.

### Clinical Assessments

The following demographic information and clinical characteristics were collected from all BSP patients: gender, education level, duration of disease, and duration of BoNT treatment. Severity of BSP was measured immediately before MRI according to the Jankovic Rating Scale (JRS; Jankovic et al., 2009) and BSP disability index (BSDI; Wabbels and Roggenkämper, 2012). The JRS includes two subscales that measure severity and frequency of involuntary orbicularis oculi muscle contraction, both based on a 5-point scale. The BSDI, a disease-specific patient-rated disability scale developed for patient self-assessment, consists of six items assessing reading, vehicle driving, shopping, watching television, walking, and doing everyday activities. Two of the six items that are most relevant to patients should be selected by the patient and a score ranging from 0% (no impairment) to 100% (not possible due to disease) reflects the severity of the impairment (Wabbels and Roggenkämper, 2012).

### Image Acquisition

Magnetic resonance images were acquired with a 3T scanner (Tim Trio; Siemens, Erlangen, Germany). The 3D T1-weighted imaging data were acquired using a magnetization-prepared rapid acquisition gradient echo pulse sequence (repetition time = 2,530 ms, echo time = 4.45 ms, inversion time = 1,100 ms, number of excitations = 1, flip angle = 7°, 192 slices, 256 × 256 matrix dimensions, 1 mm × 1 mm × 1 mm voxel size). DTI data were obtained using a single-shot echo-planar imaging sequence (64 noncollinear directions; *b* = 1,000 s/mm^2^; repetition time = 7,000 ms, echo time = 91 ms, flip angle = 90°; 128 × 128 matrix dimensions; 2 mm × 2 mm × 3 mm voxel size; 50 axial slices; 256 mm × 256 mm field of view). A reference image without diffusion weighting (b = 0 s/mm^2^) was also acquired.

### TBSS Analysis

Diffusion tensor imaging data preprocessing and analyses were performed using PANDA software (Pipeline for Analyzing Brain Diffusion Images toolkit,^1^; Cui et al., 2013). First, a binary brain mask was obtained for each subject from the respective b = 0 images. DTI were coregistered to the corresponding B0 image by affine transformations to correct eddy current distortion and head motion. The diffusion tensor matrix was reconstructed using an iterative least square algorithm to calculate FA, MD, axial diffusive (AD), and radial diffusivity (RD) maps. LDH images (pre-defined neighborhood of 27 voxels) were calculated according to our previous work (Liu et al., 2017). For between-group comparisons, the framework of TBSS was used to establish the WM correspondence between subjects (Liu et al., 2017). Specifically, non-linearly registered individual FA images in native space were transformed to Montreal Neurological Institute space and projected onto the WM skeleton. The resultant warping transformations and skeleton projections were then applied to FA, LDH, MD, AD, and RD maps. The quality of normalization was visually inspected to determine whether the normalization was adequate using a slice-by-slice procedure.

### CT Analysis

CT was calculated using FreeSurfer (v.6.0.1;^2^; Fischl, 2012). All procedures were performed using the automated surface-based pipeline with the default FreeSurfer parameters, which include Talairach registration, intensity normalization, skull-stripping, WM segmentation, tessellation of the gray/white matter boundary, and pial surface generation. The distance between each pair of vertices on the gray/white matter surface and the corresponding pial surface was defined as the CT between the vertex pair. To compare CT between groups, the cortical surface of each subject was transformed into an average surface space (fsaverage, provided in FreeSurfer package).

### Statistical Analysis

Statistical differences in age and gender between groups were tested using a Mann-Whitney U and chi-square tests, respectively. Normality testing was performed before statistical comparison using the Shapiro-Wilk test. Voxel-wise permutation statistics were applied on the skeleton-space FA and LDH data using unpaired comparisons between BSP patients and healthy controls (Liu et al., 2017). The significance threshold was set to *P* < 0.05, and age and gender were included as covariates. Significant voxel clusters were labeled using the JHU ICBMDTI-81 WM atlas (Mori et al., 2005). An unpaired *t*-test was used to compare the CT differences between groups. We used surface-based permutation testing to correct for multiple comparisons and a cluster-forming threshold was set at *P* < 0.05. Age, gender, and total intracranial volume were used as covariates. Spearman’s partial correlation analyses were conducted to investigate the relationships among CT, FA, and LDH of significantly different areas obtained via whole-brain comparison and JRS and BSDI scores after adjusting for age, duration of disease, and duration of BoNT treatment as covariates. Pearson correlations were computed to indicate the degree of correlation between LDH and FA, MD, AD, and RD. The analyses were performed using SPSS (version 16.0; IBM, Armonk, NY, United States). A *P*-value < 0.05 was used to demonstrate statistical significance.

## Results

### Participant Characteristics

A total of 30 healthy controls and 32 patients with BSP were recruited; two patients were excluded from analyses due to the presence of stroke lesions and one patient refused further MRI examination due to claustrophobia. The remaining 29 patients (18 women and 11 men; median age = 56 years) were included. Demographic information and clinical assessments for both groups are detailed in Table 1. No significant differences in age, gender, and educational level were observed between groups.

**TABLE 1:** Subjects demographics and clinical assessments.

	**Patients group (*n* = 29)**	**Control group (*n* = 30)**
Median age, *y*	56 (28–75)	60 (26–75)
Women, *n* (%)	17 (58.62)	19 (63.3)
Education, *y* (range)	12 (3–16)	12 (3–17)
Median JRS (range)	6 (2–8)	
Median BSDI (range)	1 (0–1.8)	
Median duration, *y* (range)	7 (1–25)	
Median BoNT duration, *y* (range)	2 (0–20)	

*Empty cells indicate no assessment. BoNT, botulinum neurotoxin; BSDI, blepharospasm disability index; JRS, Jankovic rating scale.*

### Intergroup Differences in FA, LDH, and CT

**FIGURE 1:**
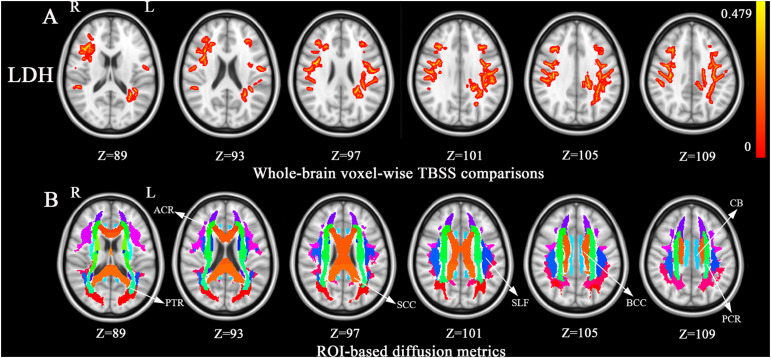
Tract-based spatial statistics (TBSS) analysis of white matter diffusion. **(A)** Differences in local diffusion homogeneity (LDH) between the patient group and the HC group. Orange (thickened for better visibility) represents increased LDH in the patient group when compared to the HC group (family-wise error [FWE], *P* < 0.05). **(B)**, The Johns Hopkins University white matter atlas was overlaid on the normalized T1-weighted images in the standard ICBM-DTI-81 space. Colored regions indicate major white matter tracts exhibiting differences between the patient and HC groups. Color bar denotes the *t*-values. Abbreviations: ACR, anterior corona radiate; BCC, the body of corpus callosum; CB, cingulum bundle; FA, fractional anisotropy; HC, healthy control; L, left; PCR, posterior corona radiata; PTR, posterior thalamic radiata; R, right; ROI, region of regions; SCC, the splenium of corpus callosum; SLF, superior longitudinal fasciculus.

Compared with controls, no significant changes in CT and FA were detected in BSP patients. As shown in Figure 1, compared to healthy controls, BSP patients showed higher LDH in four clusters including ROI A (Cohen’s *d* = 1.27), which included the left superior longitudinal fasciculus (SLF), body and splenium of the CC, left posterior corona radiata (CR), and left posterior thalamic radiata (PTR); ROI B (*d* = 1.70), which included the right SLF, right anterior and superior CR, and genu of the CC; ROI C (*d* = 1.15), which included the right SLF and right superior CR; and ROI D (*d* = 1.47), which included the left cingulum bundle and splenium of the CC. The cluster details are listed in Table 2.

**TABLE 2:** Regions showing group differences in local diffusion homogeneity.

	**Region names**	**Hemisphere**	**Voxel size**
ROI A	Superior longitudinal fasciculus	Left	1068
	Splenium of corpus callosum		228
	Posterior corona radiata	Left	183
	Posterior thalamic radiation	Left	117
	Body of corpus callosum		102
ROI B	Superior longitudinal fasciculus	Right	286
	Anterior corona radiata	Right	172
	Superior corona radiata	Right	15
	Genu of corpus callosum		8
ROI C	Superior longitudinal fasciculus	Right	430
	Superior corona radiata	Right	9
ROI D	Cingulum (cingulate gyrus)	Left	46
	Splenium of corpus callosum		4

*ROI represents region of interest.*

### Correlation Analyses

**FIGURE 2:**
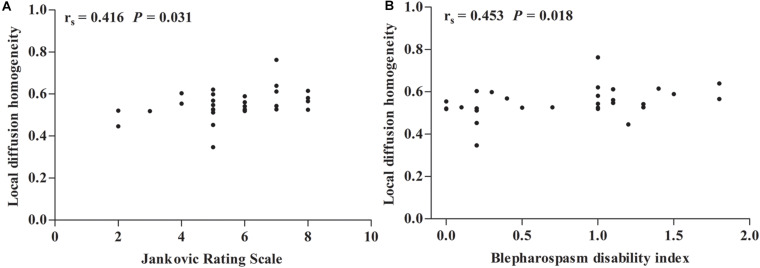
Spearman correlation maps. **(A,B)** Spearman correlation plots for local diffusion homogeneity in the region of interest (ROI) A (*y*-axis), as well as Jankovic rating scale and blepharospasm disability index (*x*-axis). ROI A represents left superior longitudinal fasciculus, splenium of corpus callosum, posterior corona radiata, posterior thalamic radiation (include optic radiation), body of corpus callosum, and superior corona radiata. *r*_*s*_ = Spearman correlation coefficient.

As shown in Figure 2, Spearman’s partial correlation analysis revealed that LDH values in ROI A were positively correlated with JRS sum scores (*r*_*s*_ = 0.416, *P* = 0.031) and BSDI (*r*_*s*_ = 0.453, *P* = 0.018) when adjusting for age, duration of disease, and duration of BoNT treatment as covariates in patients with BSP. There were no significant correlations between LDH values in other ROIs and JRS and BSDI scores. The Pearson correlation analysis indicated that there were no significant corrections between LDH and FA, MD, AD, and RD (all *P* > 0.05).

## Discussion

### WM Changes in BSP Patients

In the present study, we found that BSP patients demonstrated increased LDH in multiple WM regions, and LDH values in the left SLF, body and splenium of the CC, posterior CR, and PTR were positively correlated with severity of BSP; however, no differences in FA were found between BSP patients and healthy controls. These findings suggest that each of these paraments are differentially sensitive to detecting BSP-related WM abnormalities and LDH may be useful in assessing disability in patients with BSP.

We did not observe any significant differences in FA values between groups in the TBSS analysis, which is consistent with evidence from the majority of DTI studies (Fabbrini et al., 2008; Horovitz et al., 2012). However, our findings contradict one DTI study assessing BSP patients using Statistical Parametric Mapping (SPM)-based statistical analysis, which reported significant FA decrease in the left anterior lobe of the cerebellum (Yang et al., 2014). Differences in imaging methodologies across studies may account for these discrepancies and inaccurate registration of DTI-derived maps has been highlighted as a concern in the SPM-based approach. In addition, aggravating the partial volume problem caused by smoothing may result in inaccurate FA changes with the SPM-based approach. Conversely, TBSS avoids issues of registration and smoothing of diffusion data.

In contrast to FA and LDH was significantly increased in the SLF, CC, CR, and thalamic radiata in BSP patients, suggesting that WM abnormalities are present in patients with BSP. Several factors may be responsible for such differences. FA and LDH are two different types of diffusion metrics and have different sensitivities to specific WM microstructural properties. FA reflects intra-voxel diffusion shape, whereas LDH, an inter-voxel diffusion measure, reflects the microstructural coherence changes of the underlying WM fiber bundles (Alexander et al., 2007). In addition, the SLF, CC, CR, and thalamic radiata contain complex WM tracts, including crossing fibers. FA may be substantially affected by crossing fibers, whereas LDH is a model-free index that is better tolerates fiber crossing (Liu et al., 2016, 2017).

### Increased LDH Indicates Compensatory Effects

Increased LDH represents the increments in the local coherence of WM fiber tracts. We interpreted increases in LDH as consequences of an early neuroplasticity-induced compensatory mechanism (Gong, 2013; Liang et al., 2019). Moreover, significant correlations between increased LDH values in the left SLF, body and splenium of the CC, left posterior CR, and left PTR and disease severity in BSP patients further confirmed our interpretation.

### SLF

The SLF is a pair of bidirectional association fibers that connect the occipital, parietal, temporal, and frontal lobes. The SLF is an important structure and is considered critical for the regulation of spatial attention, motor behavior, and visual oculomotor functions, articulation of nature language, transfer of somatosensory signals between motor and parietal cortices, and integration of auditory information (Merchant, 2018). In BSP, the critical role of abnormalities in the integration of sensory input with control of motor output at the brain stem, basal ganglionic, and sensorimotor cortical level has been highlighted by recent neurophysiological studies (Martino et al., 2011). Therefore, the abnormalities in circuits for sensory input processing and integration of sensory input with motor output in BSP pathophysiology may be compensated by the increased LDH in the SLF for maintaining typical neural functions.

### CR

Corona radiata fibers are projection fibers that connect the cortex to the brainstem and thalamus in afferent and efferent manners (Morecraft et al., 2017). Lesions in the brainstem and thalamus resulting in BSP have been widely reported (Jankovic and Patel, 1983; Khooshnoodi et al., 2013). Obermann et al. (2007) proposed that compensatory neuronal hyperactivity could occur due to structural lesions of the brain in BSP. Moreover, it has been suggested that hyperactivity in the basal ganglia-thalamo-cortical motor circuit may lead to dystonia (Suzuki et al., 2007). Baker et al. (2003) reported a larger activation in the superior cerebellum, anterior cingulate cortex, anterior visual cortex, central region of the thalamus, and primary motor cortex during voluntary blinking in BSP patients compared to controls. They attributed their findings to a hyperactive cortical circuit connecting the cerebellum, supplementary motor cortex, limbic system, visual cortex, and supranuclear motor fibers innervating the periorbital muscles. Suzuki et al. (2011) observed gray matter density increases in the bilateral primary sensorimotor cortices in patients with BSP, which were dependent on the strength and duration of BSP. They suggested that an increase of gray matter density in the bilateral primary sensorimotor cortices may be secondary to long-term hyperactivity in these areas. Therefore, the documented increase in LDH in the CR in our current study may reflect an anatomical substrate for these functional findings arising from compensatory neuronal hyperactivity.

### CC and PTR

Previous studies have indicated that the CC integrates and transfers information from both cerebral hemispheres to process high-level cognitive signals (Musiek, 1986; Edwards et al., 2014). The PTR projects to the occipital, temporal, and parietal cortices; it is thereby connected to cortical regions involved in the processing of visual and cognitive functions. The observed increase in LDH in the splenium of the CC and PTR in BSP is difficult to explain due to the absence of direct involvement of these tracts in motor control. Notably, patients with BSP have been found to perform worse than the controls on complex movement planning, motor dexterity, visuospatial working memory, tactile object recognition, and sustained attention (Bugalho et al., 2008; Aleman et al., 2009). Whether some of the CC and PTR abnormalities detected by LDH reflect compensatory changes for impairments in nonmotor (e.g., cognitive) aspects of BSP requires further exploration.

### CT Changes in BSP Patients

Here, we did not observe any differences in CT between BSP patients and controls. Our findings contradict two CT studies assessing BSP patients, which reported widespread cortical atrophy in motor, sensory, and visual processing regions compared to healthy controls (Hanganu et al., 2016; Vilany et al., 2017); however, an uncorrected *P*-value was used to identify regions with significant CT changes in these studies, which may increase the rate of false positives. Changes in gray matter volume have been found in widespread cortical areas using VBM in BSP patients (Etgen et al., 2006; Obermann et al., 2007; Martino et al., 2011; Suzuki et al., 2011; Horovitz et al., 2012). Changes in CT, surface area, and gyrification may mediate changes in gray matter volume (Kong et al., 2015). Therefore, our findings suggest that gray matter volume changes reported in previous VBM studies are unlikely to be driven by CT changes.

### Limitations

Some limitations in the current study should be discussed. First, the true biological relevance of LDH remains unclear. Although we have proposed tentative interpretations regarding the potential neural mechanisms and biological substrates for LDH, these interpretations remain speculative. Second, although potential influences of BoNT on DTI metrics remain unclear, these concerns should be considered. Further studies should be conducted in BSP patients without BoNT treatment. Thirdly, the TBSS-based method is insensitive to FA changes outside the local centers of WM bundles. Furthermore, since each voxel is projected to the nearest tract center location, areas centered between the two skeleton points can be artificially divided into multiple anatomical locations, making our findings difficult to explain because our results may be driven by other voxels (Schwarz et al., 2014). Finally, in the present study, there were four BSP patients (13.8%) who were younger than 40 years of age, which is slightly higher than the 5 and 12.3% reported by two previous studies of 111 and 57 patients with BSP, respectively (Jankovic and Ford, 1983; Hwang, 2012). The relatively small sample size, we believe, may partly account for the disproportion of younger patients. Thus, future studies with larger sample sizes are needed to refine our current findings.

## Conclusion

In conclusion, we found that intra-voxel (FA) and inter-voxel (LDH) diffusive parameters are differentially sensitive to detecting BSP-related WM abnormalities. We observed notable compensatory recruitment of the left SLF, body and splenium of the CC, left posterior CR, and left PTR with increased LDH in BSP compared to healthy controls. These compensatory mechanisms and potential relationships with disease severity might be useful for the assessment of disability in BSP patients.

## Data Availability Statement

The datasets for this article are not publicly available because of participant privacy. Requests to access the datasets should be directed to GL, liug26@mail.sysu.edu.cn.

## Ethics Statement

The studies involving human participants were reviewed and approved by the First Affiliated Hospital of Sun Yat-sen University Clinical Research Review Board. The patients/participants provided their written informed consent to participate in this study.

## Author Contributions

YG performed the experimental work and wrote the manuscript. KP, LZ, and CX performed the experimental work and assisted in the statistical analysis. ZO and YW assisted in selecting patients and summarized the clinical tables. WZ designed the experimental work and assisted in selecting patients. JZ designed the experimental work and edited the manuscript. GL coordinated the design of this study, designed the experimental work, and wrote and edited the manuscript. All authors contributed to the article and approved the submitted version.

## Conflict of Interest

The authors declare that the research was conducted in the absence of any commercial or financial relationships that could be construed as a potential conflict of interest.

## Footnotes

1 http://www.nitrc.org/projects/panda/

2 http://surfer.nmr.mgh.harvard.edu/

## References

[B1] Albanese A. Bhatia K. Bressman S. B. Delong M. R. Fahn S. Fung V. S. (2013). Phenomenology and classification of dystonia: a consensus update. *Mov. Disord.* 28 863–873. 10.1002/mds.25475 23649720 PMC3729880

[B2] Aleman G. G. de Erausquin G. A. Micheli F. (2009). Cognitive disturbances in primary blepharospasm. *Mov. Disord.* 24 2112–2120. 10.1002/mds.22736 19705473

[B3] Alexander A. L. Lee J. E. Lazar M. Field A. S. (2007). Diffusion tensor imaging of the brain. *Neurotherapeutics* 4 316–329. 10.1016/j.nurt.2007.05.011 17599699 PMC2041910

[B4] Baker R. S. Andersen A. H. Morecraft R. J. Smith C. D. (2003). A functional magnetic resonance imaging study in patients with benign essential blepharospasm. *J. Neuroophthalmol.* 23 11–15. 10.1097/00041327-200303000-00003 12616082

[B5] Berman B. D. Honce J. M. Shelton E. Sillau S. H. Nagae L. M. (2018). Isolated focal dystonia phenotypes are associated with distinct patterns of altered microstructure. *Neuroimage Clin.* 19 805–812. 10.1016/j.nicl.2018.06.004 30013924 PMC6024227

[B6] Bugalho P. Corrêa B. Guimarães J. Xavier M. (2008). Set-shifting and behavioral dysfunction in primary focal dystonia. *Mov. Disord.* 23 200–206. 10.1002/mds.21784 18044708

[B7] Cui Z. Zhong S. Xu P. He Y. Gong G. (2013). PANDA: a pipeline toolbox for analyzing brain diffusion images. *Front. Hum. Neurosci.* 7:42. 10.3389/fnhum.2013.00042 23439846 PMC3578208

[B8] Defazio G. Hallett M. Jinnah H. A. Conte A. Berardelli A. (2017). Blepharospasm 40 years later. *Mov. Disord.* 32 498–509. 10.1002/mds.26934 28186662 PMC5941939

[B9] Edwards T. J. Sherr E. H. Barkovich A. J. Richards L. J. (2014). Clinical, genetic and imaging findings identify new causes for corpus callosum development syndromes. *Brain* 137 1579–1613. 10.1093/awt358 24477430 PMC4032094

[B10] Etgen T. Muhlau M. Gaser C. Sander D. (2006). Bilateral grey-matter increase in the putamen in primary blepharospasm. *J. Neurol. Neurosurg. Psychiatry.* 77 1017–1020. 10.1136/jnnp.2005.087148 16690695 PMC2077759

[B11] Fabbrini G. Pantano P. Totaro P. Calistri V. Colosimo C. Carmellini M. (2008). Diffusion tensor imaging in patients with primary cervical dystonia and in patients with blepharospasm. *Eur. J. Neurol.* 15 185–189. 10.1111/j.1468-1331.2007.02034.x 18217887

[B12] Fischl B. (2012). FreeSurfer. *Neuroimage* 62 774–781. 10.1016/j.neuroimage.2012.01.021 22248573 PMC3685476

[B13] Gong G. (2013). Local diffusion homogeneity (LDH): an inter-voxel diffusion MRI metric for assessing inter-subject white matter variability. *PLoS One* 8:e66366. 10.1371/journal.pone.0066366 23776665 PMC3679045

[B14] Hamilton M. (1959). The assessment of anxiety states by rating. *Br. J. Med. Psychol.* 32 50–55. 10.1111/j.2044-8341.1959.tb00467.x 13638508

[B15] Hanganu A. Muthuraman M. Chirumamilla V. C. Koirala N. Paktas B. Deuschl G. (2016). Grey matter microstructural integrity alterations in blepharospasm are partially reversed by botulinum neurotoxin therapy. *PLoS One* 11:e0168652. 10.1371/journal.pone.0168652 27992533 PMC5161386

[B16] Horovitz S. G. Ford A. Najee-Ullah M. A. Ostuni J. L. Hallett M. (2012). Anatomical correlates of blepharospasm. *Transl Neurodegener.* 1:12. 10.1186/2047-9158-1-12 23210426 PMC3514098

[B17] Hwang W. J. (2012). Demographic and clinical features of patients with blepharospasm in southern Taiwan: a university hospital-based study. *Acta Neurol Taiwan.* 21 108–114. 23196730

[B18] Jankovic J. Ford J. (1983). Blepharospasm and orofacial-cervical dystonia clinical and pharmacological findings in 100 patients. *Ann. Neurol.* 13 402–411. 10.1002/ana.410130406 6838174

[B19] Jankovic J. Kenney C. Grafe S. Goertelmeyer R. Comes G. (2009). Relationship between various clinical outcome assessments in patients with blepharospasm. *Mov. Disord.* 24 407–413. 10.1002/mds.22368 19053054

[B20] Jankovic J. Patel S. C. (1983). Blepharospasm associated with brainstem lesions. *Neurology* 33 1237–1240. 10.1212/wnl.33.9.1237 6684264

[B21] Khooshnoodi M. A. Factor S. A. Jinnah H. A. (2013). Secondary blepharospasm associated with structural lesions of the brain. *J. Neurol. Sci.* 331 98–101. 10.1016/j.jns.2013.05.022 23747003 PMC3732185

[B22] Kong L. Herold C. J. Zöllner F. Salat D. H. Lässer M. M. Schmid L. A. (2015). Comparison of grey matter volume and thickness for analysing cortical changes in chronic schizophrenia: a matter of surface area, grey/white matter intensity contrast, and curvature. *Psychiatry Res.* 231 176–183. 10.1016/j.pscychresns.2014.12.004 25595222

[B23] Liang Y. Zhang H. Tan X. Liu J. Qin C. Zeng H. (2019). Local diffusion homogeneity provides supplementary information in T2DM-related WM microstructural abnormality detection. *Front. Neurosci. doi* 13:63. 10.3389/fnins.2019.00063 30792623 PMC6374310

[B24] Liu G. Tan S. Dang C. Peng K. Xie C. Xing S. (2017). Motor recovery prediction with clinical assessment and local diffusion homogeneity after acute subcortical infarction. *Stroke.* 48 2121–2128. 10.1161/STROKEAHA.117.017060 28630233

[B25] Liu H. H. Wang J. Chen X. M. Li J. P. Ye W. Zheng J. (2016). Reduced local diffusion homogeneity as a biomarker for temporal lobe epilepsy. *Medicine* 95:e4032. 10.1097/MD.0000000000004032 27472676 PMC5265813

[B26] Martino D. Di Giorgio A. D’Ambrosio E. Popolizio T. Macerollo A. Livrea P. (2011). Cortical gray matter changes in primary blepharospasm: a voxel-based morphometry study. *Mov. Disord.* 26 1907–1912. 10.1002/mds.23724 21717508

[B27] Merchant R. E. (2018). “Superior Longitudinal Fasciculus,” in *Encyclopedia of Clinical Neuropsychology*, eds Kreutzer J. DeLuca J. Caplan B. (Cham: Springer).

[B28] Morecraft R. J. Binneboese A. Stilwell-Morecraft K. S. Ge J. (2017). Localization of orofacial representation in the corona radiata, internal capsule and cerebral peduncle in *Macaca mulatta*. *J. Comp. Neurol.* 525 3429–3457. 10.1002/cne.24275 28675473 PMC5861721

[B29] Mori S. Wakana S. Nagae-Poetscher L. M. van Zijl P. C. M. (2005). *MRI Atlas of Human White Matter.* Amsterdam: Elsevier Science.

[B30] Musiek F. E. (1986). Neuroanatomy, neurophysiology, and central auditory assessment. Part III: corpus callosum and efferent pathways. *Ear Hear.* 7 349–358. 10.1097/00003446-198612000-00001 3792676

[B31] Obermann M. Yaldizli O. De Greiff A. Lachenmayer M. L. Buhl A. R. Tumczak F. (2007). Morphometric changes of sensorimotor structures in focal dystonia. *Mov. Disord.* 22 1117–1123. 10.1002/mds.21495 17443700

[B32] Schmidt K. E. Linden D. E. Goebel R. Zanella F. E. Lanfermann H. Zubcov A. A. (2003). Striatal activation during blepharospasm revealed by fMRI. *Neurology* 60 1738–1743. 10.1212/01.wnl.0000063306.67984.8c 12796523

[B33] Schwarz C. G. Reid R. I. Gunter J. L. Senjem M. L. Przybelski S. A. Zuk S. M. (2014). Improved DTI registration allows voxel-based analysis that outperforms tract-based spatial statistics. *Neuroimage* 94 65–78. 10.1016/j.neuroimage.2014.03.026 24650605 PMC4137565

[B34] Suzuki Y. Kiyosawa M. Wakakura M. Mochizuki M. Ishii K. (2011). Gray matter density increase in the primary sensorimotor cortex in long-term essential blepharospasm. *Neuroimage* 56 1–7. 10.1016/j.neuroimage.2011.01.081 21310245

[B35] Suzuki Y. Mizoguchi S. Kiyosawa M. Mochizuki M. Ishiwata K. Wakakura M. (2007). Glucose hypermetabolism in the thalamus of patients with essential blepharospasm. *J. Neurol.* 254 890–896. 10.1007/s00415-006-0468-5 17325818

[B36] Vilany L. de Rezende T. J. R. Piovesana L. G. Campos L. S. de Azevedo P. C. Torres F. R. (2017). Exploratory structural assessment in craniocervical dystonia: global and differential analyses. *PLoS One* 12:e0182735. 10.1371/journal.pone.0182735 28829782 PMC5567646

[B37] Wabbels B. Roggenkämper P. (2012). Botulinum toxin in hemifacial spasm: the challenge to assess the effect of treatment. *J. Neural. Transm.* 119 963–980. 10.1007/s00702-011-0762-y 22231846

[B38] Yang J. Luo C. Song W. Guo X. Zhao B. Chen X. (2014). Diffusion tensor imaging in blepharospasm and blepharospasm-oromandibular dystonia. *J. Neurol.* 261 1413–1424. 10.1007/s00415-014-7359-y 24792726

